# Clinical Considerations of *BRCA1*- and *BRCA2*-Mutation Carriers: A Review

**DOI:** 10.1155/2011/374012

**Published:** 2011-08-08

**Authors:** O. Bougie, J. I. Weberpals

**Affiliations:** ^1^The Department of Obstetrics, Gynaecology, and Newborn Care, The Ottawa Hospital, Ottawa, ON, Canada K1H 8L6; ^2^Centre for Cancer Therapeutics, Ottawa Hospital Research Institute, The Ottawa Hospital, Ottawa, ON, Canada K1H 8L6; ^3^The Division of Gynaecologic Oncology, The Ottawa Hospital, Ottawa, ON, Canada K1H 8L6

## Abstract

Individuals who carry an inherited mutation in the breast cancer 1 (*BRCA1*) and *BRCA2* genes have a significant risk of developing breast and ovarian cancer over the course of their lifetime. As a result, there are important considerations for the clinician in the counseling, followup and management of mutation carriers. This review outlines salient aspects in the approach to patients at high risk of developing breast and ovarian cancer, including criteria for genetic testing, screening guidelines, surgical prophylaxis, and chemoprevention.

## 1. Introduction

Research into the hereditary breast and ovarian cancer genes, breast cancer 1 (*BRCA1*) and *BRCA2*, is an area of ongoing discovery in the molecular biology of cancer. For clinicians, it can be an overwhelming challenge to keep a breast with the latest studies and incorporate the most up-to-date evidence into their practice. There are many unique considerations that should be addressed when approaching patients with a predisposition to hereditary breast and ovarian cancer, including counseling, screening, and risk-reducing strategies. It is also important to understand which high-risk patients should be referred for genetic counseling for consideration of *BRCA1/2* gene testing, an approach which is often underutilized in physicians' practices [1]. In this review, the most relevant and current studies in hereditary breast and ovarian cancer epidemiology, screening, and prevention are outlined to guide the clinician in the management of high-risk individuals. Also highlighted is the role of BRCA in sporadic breast and ovarian cancer and the emergence of novel targeted therapies such as poly(ADP-ribose) polymerase inhibitors (PARPi) in BRCA1-deficient patients.

## 2. Cancer Risk in *BRCA1*- and *BRCA2*- Mutation Carriers

In the general population, a woman's lifetime risk of breast cancer is 1 in 8 [2], and the risk of ovarian cancer is 1 in 70 [3]. It is estimated that a *BRCA1/2* mutation is found in 2–6% of breast cancer patients [4–6] and 10–15% of epithelial ovarian cancer patients [7–9]. In all women, the prevalence of a *BRCA1* mutation is 1 in 800 to 1 in 1400 and the prevalence of *BRCA2* mutation is slightly lower at 1 in 450 to 1 in 800 [6, 10, 11]. The lifetime risk of breast cancer in *BRCA1- *and* BRCA2-*mutation carriers is 45–80% [12, 13]. The lifetime risk of ovarian cancer is 45–60% for *BRCA1-*mutation carriers and 11–35% for *BRCA2*-mutation carriers [12–14]. A number of studies examining the incidence of breast cancer have reported a lower likelihood amongst *BRCA2*-mutation carriers compared to *BRCA1-*mutation carriers [13, 15]. Among the Ashkenazi Jewish population, the prevalence of a *BRCA1/2* mutation is as high as 2.5% [16]. In breast cancer patients of Jewish origin, the *BRCA1 *mutation rate is increased at 10% compared to the general breast cancer population [16]. Even more significant, among Jewish woman with ovarian cancer, there is an estimated 29% risk of carrying a *BRCA1 *or* BRCA2* mutation [17]. 

Cancer risk has been shown to increase substantially with each decade of life for *BRCA1- *and *BRCA2*-mutation carriers [12]. A woman with a *BRCA* mutation has a 20% chance of developing breast cancer by the time she is 40 years old. However, the risk increases to 37% by the age of 50, 55% by the age of 60, and is over 70% by the age of 70. For *BRCA1*-mutation carriers, the risk of ovarian cancer rises sharply in the 4th decade of life and then increases progressively by approximately 10% with each subsequent decade of life. 

Individuals who have a known *BRCA* mutation are also at risk for other malignancies. Approximately 30% of women with fallopian tube cancer have a mutation in *BRCA1 *or* BRCA2* [18]. In addition, the inheritance of a *BRCA1* mutation has been linked with an increased risk of endometrial, pancreatic, and prostate cancer [19, 20]. Specifically, the increased risk of prostate cancer is associated with the *BRCA1* 4153delA and the C61G mutations [21]. A *BRCA1* mutation has been shown to be associated with an increased risk of early onset colon cancer in a defined population but does not seem to increase the overall risk of colon cancer [22]. Patients with a *BRCA2* mutation have an increased risk of prostate and pancreatic cancer [23–25]. An earlier study by the Breast Linkage Consortium reported an increased incidence of gallbladder, bile duct, stomach cancer, and malignant melanoma in *BRCA2*-mutation carriers, yet recent evidence has not substantiated this association [26]. Only the study by Debniak et al. [27] demonstrated that one common variant of the *BRCA2* gene, the N991D variant, is linked with an increased malignant melanoma risk.

## 3. Clinicopathologic Features of Hereditary Breast and Ovarian Cancer

There are several distinguishing clinical and pathologic features of breast and ovarian cancer related to *BRCA* mutation status. The features of hereditary breast and ovarian cancers are summarized in Table 1. Compared to nonhereditary breast cancer patients, *BRCA1-*mutation carriers are diagnosed with breast cancer at a younger age [18, 28], and up to 80% of cancers occur prior to menopause [18, 29]. At initial presentation, hereditary breast cancer patients have a higher incidence of bilateral breast cancer, poorly differentiated tumours, and lymph node positivity [18]. BRCA1-associated tumours are more often estrogen receptor (ER) [18], progesterone receptor (PR) [30], and human epidermal growth factor 2 (HER2) negative compared to their sporadic counterparts [31]. BRCA2 tumours, in turn, demonstrate more ER positivity [18], while the rate of PR and HER2 receptor positivity is similar to that of sporadic tumours [30]. BRCA1 tumours are more likely to be p53 positive, while BRCA2 tumours are usually p53 negative [32]. Lee et al. [33] conducted a recent systemic review to assess the survival pattern of breast cancer patients with *BRCA1* and *BRCA2* mutations and found that the *BRCA1*-mutation carriers had a significantly decreased progression-free as well as overall survival compared to non-*BRCA1*-associated breast cancer patients, while *BRCA2* mutations carriers had survival patterns comparable to the general breast cancer patients. There is emerging evidence to suggest that *BRCA1*-linked breast cancer patients treated with chemotherapy regimens containing platinum or doxorubicin and cyclophosphamide have a high likelihood of achieving pathologic complete remission [34]. 

*BRCA1*-mutation carriers are diagnosed with ovarian cancer at a younger age (average age 52) compared to *BRCA2*-mutation carriers (age 62) and sporadic cases (age 63) [35]. In contrast to breast cancer, *BRCA*-associated and sporadic ovarian tumours have been reported to have similar clinicopathological features (histology, grade, and stage at diagnosis) [35]. While Boyd et al. [35] found comparable ovarian tumour histology between BRCA-associated and sporadic ovarian tumours, Piek et al. [36] found a higher incidence of papillary serous histology among BRCA-associated ovarian tumours. Patients with *BRCA1*- or *BRCA2*-linked ovarian cancers were shown to have a longer disease-free interval following chemotherapy and improved survival compared to patients with sporadic ovarian cancer ovarian cancer [35, 37, 38].

## 4. Pathophysiology of *BRCA*-Mutations

*BRCA1* and *BRCA2* are tumour suppressor genes located on chromosome 17q21 [39] and 13q12-13 [40], respectively. Hundreds of different mutations can occur within a gene leading to protein inactivation, with the genetic defect resulting most commonly in a truncated protein product. However, a number of missense mutations and large gene rearrangements can also cause a mutant phenotype [41]. Mutation types tend to correspond with various populations and ethnicities. For instance, in African Americans, 25% of *BRCA1 *mutations were frameshift, 38% missense, 13% nonsense, and 25% splice mutations, while in non-Hispanic white patients, 36% of *BRCA1* mutations are frameshift, 14% are missense, 29% nonsense, and 21% are splice mutations [5]. In the Ashkenazi Jewish population, there are three distinct mutations in *BRCA1* and* BRCA2* genes that are responsible for the majority of the *BRCA* mutations seen in this population; namely, these are the *BRCA1* 185delAG, *BRCA1 *5382insC, and *BRCA2* 6174delT mutations [42]. 

The process of carcinogenesis is often initiated by repeated episodes of DNA damage, which can occur secondary to a number of stressors, such as reactive oxygen species, cytotoxic chemotherapy, and ionizing radiation. DNA double-strand breaks (DSB) are particularly harmful, as both complementary strands of DNA are damaged leading to replication arrest. BRCA1 and BRCA2 play an integral role in the response to cellular stress, the localization to sites of damaged DNA, and the activation of DNA repair processes. In particular, BRCA1 and BRCA2 usually repair DSB through the conservative mechanism of homologous recombination [43–45]. Central to the process of homologous recombination, the BRCA proteins bind an essential recombinant, RAD51, and mutations affecting the binding ability of BRCA1/2 to RAD51, which lead to genomic instability [45–47]. While BRCA2 is primarily involved in homologous recombination repair, BRCA1 is involved in alternate DNA repair processes such as nonhomologous end joining, which is error prone [48]. *BRCA* deficiency ultimately leads to the accumulation of genetic alterations as a result of the failure of cells to arrest and repair DNA damage or to self-destruct, resulting in neoplastic progression.

## 5. Genetic Testing

Identifying patients that should be considered for genetic testing is an important aspect of practice that is often overlooked [1]. Pedigree analysis can be used in conjunction with available risk assessment models to determine whether a family is suspected of having hereditary, familial, or sporadic cancer [49]. *BRCA1 *or* BRCA2* gene mutations are inherited in an autosomal dominant fashion. The Manchester scoring system is a risk assessment model which may be applied to evaluate an individual's risk of having a *BRCA1 *or* BRCA2* mutation [50]. The scoring system considers the age of cancer diagnosis as well as a family history of female breast, male breast, ovarian, prostate, and pancreatic cancer in the risk stratification model. Recently, breast cancer pathology has been included in this model to increase predictive accuracy. It is considered the most sensitive model in predicting mutations in both *BRCA* genes [49, 50]. The disadvantage of the model is that it has been shown to lead to over-referral for genetic testing. 

Specific criteria for referral for genetic testing vary amongst institutions but are all based on clinical characteristics that increase one's chance of developing a hereditary breast and/or ovarian cancer. Clinical features that should prompt one to consider a genetic counseling referral are outlined in Table 2 [49]. It is important to include genetic counseling in the cancer risk assessment process [51]. A number of reports propose criteria for referral for genetic counseling regarding hereditary risk of breast and/or ovarian cancer, including the National Comprehensive Cancer Network (NCCN) [52], the U.S. Preventive Services Task Force (USPSTF) [53], American Congress of Obstetricians and Gynecologists (ACOG) [54], the National Institute for Health and Clinical Excellence (NICE) [55], and the European Society for Medical Oncology (ESMO) [56]. Guidelines in most countries use a 10–20% threshold of detecting a mutation in *BRCA1/2* within a given family before mutational analysis is considered. A recent study analyzed the cost effectiveness of screening for cancer susceptibility genes and reported that when one's risk of having a mutation is at only 10%, genetic screening is not cost effective [57].

## 6. Surveillance

### 6.1. Breast Cancer

The NCCN recommends that breast cancer screening for *BRCA1- *and* BRCA2*-mutation carriers should include annual mammography and clinical breast examination every 6–12 months, starting at age 25 or individualized based on one's family history [52]. In Europe, ESMO also recommends screening with annual mammography and MRI starting at age 25–30 [56]. Ultrasound is generally not recommended as part of a screening algorithm but is utilized when mammography or MRI reveals abnormalities. The addition of ultrasound to screening mammography will yield an additional 1.1 to 7.2 cancers per 1000 high-risk women but will also substantially increase the number of false positive tests [58].

Several studies have confirmed that MRI has a higher sensitivity for the detection of invasive breast cancer compared to conventional screening [59, 60], and this has been reported to be most applicable to *BRCA1/2*-mutation carriers [59]. Rijnsburger et al. [60] found that 45.8% of breast cancers in *BRCA-*mutation carriers and 30.8% of breast cancers in *BRCA2-*mutation carriers were detected only by MRI. However, MRI specificity for cancer detection is lower than that of conventional screening, thereby resulting in more unnecessary biopsies [61]. Thus, it may be prudent to use MRI in conjunction with conventional imaging in screening women with a high genetic risk of breast cancer. In the study by Trecate et al. [61], it was advised to consider ultrasound evaluation when MRI yielded the only positive diagnostic result. 

Lord et al. [62] conducted a systematic review to assess the effectiveness of adding MRI to mammography with or without ultrasound and clinical examination in screening young women at high risk for breast cancer. Consistent evidence in a number of studies suggests that adding MRI provides a highly sensitive screening strategy (sensitivity range: 93–100%) compared to mammography alone (25–59%), or mammography and ultrasound with or without clinical examination (49–67%). In considering the use of MRI, there are no studies which have assessed whether MRI surveillance reduces the rates of advanced breast cancer or mortality. Thus, the effectiveness of MRI on improved survival stems from the assumption that earlier detection improves survival.

### 6.2. Ovarian Cancer

To date, no effective screening strategy has been found for either sporadic or hereditary ovarian cancer. At this time, the NCCN [52] and ACOG [54] guidelines suggest that for patients with *BRCA1 *or* BRCA2* mutations, pelvic ultrasound and CA125 every 6 months may be considered for the detection of early ovarian cancer. It is recommended that screening be initiated at age 35 or 5–10 years prior to the age of the earliest ovarian cancer diagnosis in an individual's family. 

Studies which have examined ovarian cancer screening specifically in *BRCA1/2*-mutation carriers did not find that pelvic ultrasound and CA125 were effective as a screening method [63–66]. In a retrospective audit of 341 asymptomatic, high-risk women enrolled for ovarian cancer screening, 31 women underwent explorative surgery because of abnormal findings at surveillance. At surgery, 3 cancers were diagnosed (2 ovarian, 1 endometrial) and 28 women were cancer free, suggesting that the diagnostic yield in screening women with a hereditary predisposition is very low [65]. The largest of the ovarian cancer screening studies for *BRCA*-mutation carrier studies included 981 women with *BRCA1/2* mutations who underwent screening with annual pelvic ultrasound and CA125 [63]. The study concluded that there was no improvement in survival in the cohort of patients that underwent screening when compared to an unscreened population.

Importantly, women should be made aware of symptomatology associated with ovarian cancer which should prompt medical evaluation. Symptoms associated with ovarian cancer include abdominal bloating, urinary urgency and/or frequency, increased abdominal girth, and early satiety [67]. Goff et al. attempted to develop a symptom index for the diagnosis of ovarian cancer by using the symptoms previously described with a time frame of symptoms occurring more than 12 days per month and less than 1 year in duration. The symptom index shows promise for advanced stage ovarian cancer diagnosis, with a sensitivity of 79.5% and specificity nearing 90%. However, for the detection of early stage disease, the sensitivity value was only 56.7%.

## 7. Surgical Prophylaxis

Women with a known *BRCA1 *or* BRCA2* mutation should be offered prophylactic mastectomy and bilateral salpingo-oophorectomy (BSO) in order to decrease the risk of breast and ovarian cancer. Bilateral mastectomy decreases breast cancer risk by 90% [68, 69], and BSO decreases the relative risk of ovarian and fallopian tube cancer by 80% [70, 71]. However, BSO does not affect the risk of primary peritoneal cancer [71]. BSO also leads to a reduction of estrogen stimulation on breast tissue, leading to a 50% relative risk reduction in the development of breast cancer [71]. The NCCN recommendation is that mutation carriers are offered BSO by age 40 or after they have completed childbearing [52]. Similarly, ESMO recommends prophylactic BSO after age 35 and when childbearing decisions are complete [56]. Overall, counseling on risk-reducing surgery should take into account the cumulative risk of developing breast and ovarian cancer with each decade of life as well as a woman's reproductive plans. 

To quantify the protective effect of risk-reducing surgery, Domchek et al. [72] completed a large prospective, multicenter cohort study which included 2482 women with *BRCA1 *or *BRCA2* mutations ascertained between 1974 and 2008. There were no breast cancers diagnosed in the 247 women with prophylactic mastectomy compared with 98 breast cancers diagnosed in 1372 women who did not undergo a mastectomy. Compared with women who did not undergo risk-reducing BSO, undergoing BSO was associated with lower all-cause mortality (10% versus 3%; HR, 0.40 [95% CI, 0.26–0.61]). In high risk women who are diagnosed with breast cancer, a contralateral prophylactic mastectomy in addition to the therapeutic mastectomy is associated with a significant survival advantage [73]. It has been reported that only approximately 20–25% of eligible patients choose to proceed with a prophylactic mastectomy and 50–55% undergo prophylactic BSO [74, 75]. Predictors of prophylactic surgeries were age below 60 years, personal history of prior breast cancer and a history of either mastectomy or BSO [74]. BSO was more common among women younger than 40 and among parous women [75].

To compare the effectiveness of prophylactic surgery versus breast screening on the reduction of cancer mortality, Kurian et al. [76] developed a unique computational model. Using this model, a 25-year-old *BRCA1/2*-mutation carrier was analyzed to compare enrolment in a breast screening program with annual mammography and MRI from age 25 to 69, with prophylactic mastectomy at various ages +/− BSO at age 40 or 50. With no intervention, survival probability by age 70 was estimated at 53% for *BRCA1 *and 71% for *BRCA2* mutation carriers. The most effective single intervention for *BRCA1*-mutation carriers was a BSO at age 40, yielding a 15% absolute survival gain. For *BRCA2-*mutation carriers, the most effective single intervention was a prophylactic mastectomy, yielding a 7% survival gain if performed at age 40. The combination of prophylactic mastectomy and BSO at the age of 40 improved survival more than any single intervention, yielding a 24% and 11% survival gain for *BRCA1- *and* BRCA2*-mutation carriers, respectively. 

When choosing a surveillance option, patients must be cautioned that premalignant and malignant changes can occur in spite of normal radiological investigations. When prophylactic mastectomy samples were compared between *BRCA1*-mutation carriers and healthy controls, there was an increased incidence of pre-malignant and malignant lesions detected in *BRCA1 *prophylactic mastectomy samples (42.3 versus 5.8%; *P* < 0.001) [77]. Similarly, approximately 6% of *BRCA1* carriers and 2% of *BRCA2 *carriers who undergo prophylactic BSO will have occult carcinomas [78]. Hirst et al. [79] examined the tumours of 45 *BRCA*-mutation carriers who underwent a prophylactic BSO and discovered 5 occult malignancies. Recently, insight into the pathogenesis of hereditary of ovarian cancer has revealed that some cancers believed to be of primary ovarian origin, in fact, arise from the fallopian tube [80]. As such, Greene et al. [81] have suggested that a bilateral salpingectomy with ovarian retention might serve as a temporary measure while definitive risk-reducing surgery is being contemplated in women who have completed childbearing.

## 8. Chemoprophylaxis

### 8.1. Tamoxifen

Tamoxifen is a selective estrogen receptor modulator that has an inhibitory effect on estrogen receptors in breast tissue and a proliferative effect in the endometrium. *BRCA*-mutation carriers may be offered tamoxifen for primary prevention of breast cancer [52]. However, since *BRCA1-*mutation carriers are more likely to develop ER- breast tumours, there may be little rationale to support tamoxifen for the prevention of breast cancer in this population. The effect of chemoprophylaxis with tamoxifen in patients with a hereditary predisposition has been extrapolated from studies examining the risk of developing contralateral breast cancer in *BRCA1/2*-mutation carriers who were treated with tamoxifen after their primary breast cancer diagnosis, as well as tamoxifen use in the general population. 

The National Surgical Adjuvant Breast and Bowel Project (NSABP) initiated the breast cancer prevention trial (P-1) which enrolled 13,388 high risk patients to determine the efficacy of tamoxifen in reducing breast cancer risk [82]. This study substantiated that 20 mg per day of tamoxifen for 5 years reduced the risk of primary invasive breast cancer by 49%. Genomic analysis of *BRCA1* and *BRCA2* for 288 women who developed breast cancer was completed after entry into the trial [83]. There were 19 patients with *BRCA1* or *BRCA2* mutations. 83% of BRCA1 breast tumours were ER−, whereas 76% of BRCA2 breast tumours were ER+. It was concluded that tamoxifen reduced breast cancer incidence among *BRCA2* carriers by 62%, similar to the reduction of ER+ breast cancer among all women in the breast cancer prevention trial. In contrast, tamoxifen use did not reduce breast cancer incidence among women with inherited *BRCA1* mutations. However, the results of the study were limited by the small number of patients in the cohort. 

Gronwald et al. [84] completed a case control study looking at *BRCA1- *and* BRCA2-*mutation carriers who developed bilateral breast cancer (*n* = 285) versus those who had unilateral breast cancer (*n* = 781). The multivariate odds ratio for contralateral breast cancer associated with tamoxifen use was 0.50 for carriers of *BRCA1* mutations (95% CI, 0.30–0.85) and 0.42 for carriers of *BRCA2 *mutations (95% CI, 0.17–1.02). However, the protective effect of tamoxifen was not seen among women who had undergone a BSO (OR = 0.83; 95% CI, 0.24–2.89), although there was only a small number of patients in this subgroup. In contrast, a strong protective effect of tamoxifen was apparent among women who were premenopausal or who had undergone natural menopause (OR = 0.44; 95% CI, 0.27–0.65). This study suggests that using tamoxifen in *BRCA1/2*-mutation carriers who have not undergone a BSO may result in a decreased risk of breast cancer. It has been estimated that tamoxifen reduces breast cancer risk in *BRCA1-*mutation carriers by 13% and in *BRCA2-*mutation carriers by 27% [85]. Similar results were found with respect to the reduction of contralateral breast cancer risk in patients with a *BRCA1/2* mutation treated with tamoxifen after first diagnosis of breast cancer [86, 87].

It has been reported that only 5.5% of women with a *BRCA1 *or* BRCA2* mutation have used tamoxifen to reduce their breast cancer risk [88]. Approximately half of the population at risk relied solely on screening for the early detection of breast cancer. Patients should be counseled about the increased risk of endometrial cancer following tamoxifen treatment (RR = 11.6, *P* = 0.0004) compared to *BRCA1/2*-mutation carriers without a history of tamoxifen use [89]. Hence, the risks and benefits of prophylactic hysterectomy should be discussed with those patients. It has been reported that in *BRCA1*-associated patients with a history of breast cancer, subsequent treatment with tamoxifen does not increase ovarian cancer risk [90]. Thus, it is anticipated that primary prophylactic tamoxifen treatment would not increase ovarian cancer risk.

### 8.2. Raloxifene

The study of tamoxifen and raloxifene (STAR) examined the efficacy of tamoxifen and raloxifene to decrease breast cancer risk [91, 92]. Raloxifene is a selective estrogen receptor modulator, which is used primarily for the prevention of osteoporosis. The advantages of raloxifene are that it is as effective as tamoxifen in reducing the risk of invasive breast cancer while having a lower risk of thromboembolic events and a trend towards a lower risk of uterine cancer. However, there was a nonstatistically significant higher risk of noninvasive breast cancer observed with raloxifene. *BRCA* status was not ascertained in this study, and, thus, raloxifene efficacy has not specifically been evaluated in this patient population. 

### 8.3. Oral Contraceptive Pill (OCP)

OCP use has been found to have different effects on the lifetime risk of ovarian and breast cancer. It has generally been accepted that OCP use decreases one's risk of ovarian cancer. However, much debate has ensued on whether the OCP increases one's risk of developing breast cancer. Recently, Iodice et al. [93], have performed a meta-analysis updated to March 2010 on the association between OCP use and breast or ovarian cancer in *BRCA1/2*-mutation carriers. Based on 18 studies, a total of 2855 breast cancer cases and 1503 ovarian cancer cases were reviewed. As previously noted, OCP use at any point during one's life was associated with a 50% relative risk reduction in developing ovarian cancer for *BRCA1/2*-mutation carriers. Looking specifically at duration of OCP use, each 10-year period of OCP use resulted in a 36% relative risk reduction for the development of ovarian cancer. In the meta-analysis, there was no evidence of a significant association between OCP use and breast cancer risk (summary relative risk (SRR): 1.13; 95% CI, 0.88–1.45). Specifically, OCP formulations used before 1975 correlated with an increased risk of breast cancer (SRR: 1.47; 95% CI: 1.06, 2.04), but there was no correlation with the use of more recent formulations (SRR: 1.17; 95% 0.74,1.86). There are studies which have correlated past users of the OCP to an increased risk of breast cancer [26, 94].

Figueiredo et al. [95] examined the effect of oral contraceptives and hormone replacement therapy (HRT) on the development of contralateral breast cancer in *BRCA1/2*-mutation carriers with a history of breast cancer. OCP use was not associated with increased contralateral breast cancer risk in *BRCA2*-mutation carriers (RR = 0.82; 95% CI = 0.21–3.13), and *BRCA1-*mutaion carriers who used the OCP trended towards a greater risk of contralateral breast cancer compared to nonusers, but this risk was not significant (RR = 2.38; 95% CI = 0.72–7.83). Few women had ever used HRT and there were no significant associations found between lifetime use and contralateral breast cancer risk among *BRCA1/2*-mutation carriers and noncarriers.

## 9. Hereditary Breast and Ovarian Cancer and HRT

Known *BRCA1/2* carriers often choose to undergo a risk-reducing BSO, thereby, entering a surgical menopause at an earlier age. As such, many would benefit from HRT to help alleviate menopausal symptoms. Rebbeck et al. [96] completed a prospective cohort study looking at 462 women with *BRCA1/2* mutations to evaluate breast cancer risk after BSO with and without HRT use. They found that BSO was significantly associated with breast cancer risk reduction overall (hazard ratio [HR] = 0.40; 95% CI, 0.18 to 0.92). HRT of any type after BSO did not significantly alter the reduction in breast cancer risk (HR = 0.37; 95% CI, 0.14 to 0.96) when compared to women who have not had a BSO or used HRT. In a matched case-control study of 472 postmenopausal women with a *BRCA1 *mutation, the adjusted odds ratio for breast cancer in HRT users versus nonusers was 0.58 (95% CI = 0.35 to 0.96; *P* = 0.03) [97]. Overall, it appears that short-term HRT use does not negate the protective effect of BSO on subsequent breast cancer risk in *BRCA1/2*-mutation carriers. 

Recently, the MARIE-GENICA Consortium on Genetic Susceptibility for Menopausal Hormone Therapy Related Breast Cancer Risk [98] has investigated HRT use and breast cancer risk in 3,149 postmenopausal breast cancer patients and 5,489 controls from the two German case-control studies. The study was initiated to determine the modification of breast cancer risk associated with hormone use by a subset of genes involved in hormone metabolism and cell cycle regulation, of which *BRCA1* is included. A minor allele of *BRCA1, *which carries an amino acid substitution due to a single nucleotide polymorphism, was found to affect the interaction with both RAD51 as well as the androgen receptor [98]. Preliminary evidence trended towards an increased risk in postmenopausal breast cancer in *BRCA1* minor allele carriers on estrogen hormone therapy compared to those with the major *BRCA1 *allele genotype.

## 10. BRCAness and Sporadic Cancer

The concept of “BRCAness” has evolved to reflect the traits that some sporadic cancers share with *BRCA1/2*-mutation carriers [99]. The inactivation of BRCA1 is a relatively frequent event in sporadic ovarian cancer and has been shown to occur through a number of epigenetic mechanisms such as promoter hypermethylation and loss of heterozygosity [100]. Decreased BRCA1 expression has been found in approximately 30–40% of sporadic breast cancers [101] and 15–80% of ovarian cancers [102]. BRCA1 has recently been evaluated as a tumour biomarker in a number of malignancies including ovarian [103, 104], breast [105, 106] and nonsmall cell lung cancer [107]. 

Emerging evidence suggests that tumours may be characterized by a relative level of BRCA1 deficiency at both the mRNA and protein level, resulting in BRCA1 having a potentially broader clinical relevance as a prognostic and predictive marker for patients with sporadic disease. A study in sporadic breast cancer by Margeli's group [108] found that patients with lower BRCA1 mRNA expression who were treated with neoadjuvant chemotherapy (fluorouracil, epirubicin, and cyclophosphamide) had a lower relapse rate and longer survival when compared to patients with higher BRCA1 expression. An analysis of 70 tumours from patients with sporadic ovarian cancer for BRCA1 mRNA expression correlated low BRCA1 mRNA expression with improved survival following platinum-based chemotherapy [109]. This finding was supported by our group which associated low BRCA1 mRNA expression with improved overall survival in 51 patients with sporadic ovarian cancer [104]. Thrall et al. [103] examined BRCA1 protein expression in 230 sporadic ovarian cancers and demonstrated that compared to high BRCA1 expression, low BRCA1 expression was predictive of longer overall survival (aHR = 0.51 (95% CI 0.32–0.83). Recently, our group has showed that in optimally debulked advanced ovarian cancer patients treated with platinum-based chemotherapy, low BRCA1 protein expression was significantly associated with a better progression-free survival (PFS) (median PFS was 24.7 and 16.6 months in patients with low and high BRCA1, resp.; HR = 0.56) [110]. Such findings extend the clinical importance of BRCA in women with breast and ovarian cancer patients, and further study is needed to explore BRCA as a predictive marker to targeted therapies.

## 11. Targeted Therapies for BRCA-Deficient Tumours

While DNA defects are often a step in the process of tumourigenesis, once a cell becomes cancerous, such defects may be exploitable to enhance susceptibility to chemotherapeutic agents. It is well accepted that BRCA1 deficiency leads to the dysregulation of DNA repair pathways, which in turn renders tumour cells more vulnerable to DNA damaging agents such as platinum-based chemotherapy. Poly (ADP-ribose) polymerase inhibitors (PARPi) are a novel therapeutic option for the treatment of ovarian and breast cancers which preferentially target BRCA1-defective cells and spare those with normal function [111]. The PARP family of enzymes serves a vital role in the repair of single-stranded DNA breaks (SSBs). Normally, unrepaired SSBs lead to double-strand breaks (DSBs), which are subsequently repaired in cells with normal BRCA function. However, in cells where BRCA is nonfunctioning or deficient, DSB are left unrepaired, leading to genomic instability and cell death [111]. The use of the PARP1 and PARP2 inhibitor, olaparib, has been studied in a phase I clinical trial for the treatment of advanced breast and ovarian cancer patients with *BRCA1/2* germline mutation as well as in a phase II trial in advanced breast cancer patients with *BRCA1/2* mutations, with both studies showing promising results [112, 113]. A number of clinical trials are currently ongoing with the use of various PARP inhibitors, both as single agents and in combination with chemotherapy, for both hereditary and sporadic breast and ovarian cancer.

## 12. Conclusion

Hereditary breast and ovarian cancer patients are unique in terms of age of diagnosis, pathological features, and prognosis. Clinicians working in the area of surgical oncology should be familiar with the criteria for referral of high-risk individuals for genetic testing. *BRCA1/2-* mutation carriers should be offered risk reduction strategies, in the form of prophylactic bilateral mastectomy and/or bilateral salpingo-oophorectomy at an early age. If a patient declines surgery, appropriate screening should ensue with an understanding of the limitations that exist. Chemoprevention with tamoxifen or raloxifene is used to decrease ones risk of breast cancer, and OCPs may be used effectively to reduce the risk of ovarian cancer. New evidence suggests that BRCA has a broader role as a potential biomarker in sporadic breast/ovarian cancer for the management of this patient population. Targeted therapy with PARPi is an exciting novel treatment option being tested in clinical trials, particularly for ovarian cancer patients where chemotherapy is limited.

##  Conflict of Interests

The authors have declared that there are no conflicts of interests.

## Figures and Tables

**Table 1:** Clinicopathologic characteristics of hereditary versus sporadic breast and ovarian cancer.

	Breast cancer	Ovarian cancer
Average age of diagnosis	BRCA1 ↓	BRCA1 ↓
	BRCA2 *↔*	BRCA2 *↔*

Pathological features	↓ differentiation	*↔* grade, stage
	↑ bilateral tumours	? ↑ papillary serous serology
	↑ # lymph nodes	

Receptor status	BRCA1: ↑ ER−/PR−/HER2−	
	BRCA2: similar to sporadic tumours	

Survival	↓ progression free-survival	↑ progression-free survival
	↓ overall survival	↑ overall survival

**Table 2:** Clinical features that warrant referral for genetic testing for BRCA1/2 mutations.

(i) Early-onset breast cancer, usually defined as before age 50 or 45	
(ii) Ovarian, fallopian tube or primary peritoneal cancer	
(iii) Individuals with two or more primary breast cancers, or breast and ovarian cancer in the same individual	
(iv) Male breast cancer	
(v) Two or more individuals in the family with breast and/or ovarian cancer	
(vi) Ashkenazi Jewish ancestry	

## Abbreviations

BRCA1: Breast cancer protein 1
ER: Estrogen receptor
BSO: Bilateral salpingo-oopherectomy
OCP: Oral contraceptive pill
HRT: Hormone replacement therapy
PARPi: Poly(ADP-ribose) polymerase inhibitors
RR: Relative risk.

## References

[1] van Riel E, Wárlám-Rodenhuis CC, Verhoef S, Rutgers E, Ausems M (2010). BRCA testing of breast cancer patients: medical specialists’ referral patterns, knowledge and attitudes to genetic testing. *European Journal of Cancer Care*.

[2] American Cancer Society Breast cancer facts and figures 2009-2010.

[3] American Cancer Society Cancer facts and figures.

[4] McClain MR, Palomaki GE, Nathanson KL, Haddow JE (2005). Adjusting the estimated proportion of breast cancer cases associated with BRCA1 and BRCA2 mutations: public health implications. *Genetics in Medicine*.

[5] John EM, Miron A, Gong G (2007). Prevalence of pathogenic BRCA1 mutation carriers in 5 US racial/ethnic groups. *Journal of the American Medical Association*.

[6] Malone KE, Daling JR, Doody DR (2006). Prevalence and predictors of BRCA1 and BRCA2 mutations in a population-based study of breast cancer in White and Black American women ages 35 to 64 years. *Cancer Research*.

[7] Pal T, Permuth-Wey J, Betts JA (2005). BRCA1 and BRCA2 mutations account for a large proportion of ovarian carcinoma cases. *Cancer*.

[8] Zhang S, Royer R, Li S (2011). Frequencies of BRCA1 and BRCA2 mutations among 1,342 unselected patients with invasive ovarian cancer. *Gynecologic Oncology*.

[9] Risch HA, McLaughlin JR, Cole DEC (2006). Population BRCA1 and BRCA2 mutation frequencies and cancer penetrances: a kin-cohort study in Ontario, Canada. *Journal of the National Cancer Institute*.

[10] (2000). Prevalence and penetrance of BRCA1 and BRCA2 mutations in a population-based series of breast cancer cases. Anglian Breast Cancer Study Group. *British Journal of Cancer*.

[11] Malone KE, Daling JR, Neal C (2000). Frequency of BRCA1/BRCA2 mutations in a population-based sample of young breast carcinoma cases. *Cancer*.

[12] King MC, Marks JH, Mandell JB, New York Breast Cancer Study Group (2003). Breast and Ovarian Cancer Risks Due to Inherited Mutations in BRCA1 and BRCA2. *Science*.

[13] Antoniou A, Pharoah PDP, Narod S (2003). Average risks of breast and ovarian cancer associated with BRCA1 or BRCA2 mutations detected in case series unselected for family history: a combined analysis of 22 studies. *American Journal of Human Genetics*.

[14] van der Kolk DM, de Bock GH, Leegte BK (2010). Penetrance of breast cancer, ovarian cancer and contralateral breast cancer in BRCA1 and BRCA2 families: high cancer incidence at older age. *Breast Cancer Research and Treatment*.

[15] Warner E, Foulkes W, Goodwin P (1999). Prevalence and penetrance of BRCA1 and BRCA2 gene mutations in unselected Ashkenazi Jewish women with breast cancer. *Journal of the National Cancer Institute*.

[16] Roa BB, Boyd AA, Volcik K, Richards CS (1996). Ashkenazi jewish population frequencies for common mutations in BRCA1 and BRCA2. *Nature Genetics*.

[17] Modan B, Hartge P, Hirsh-Yechezkel G (2001). Parity, oral contraceptives, and the risk of ovarian cancer among carriers and noncarriers of a BRCA1 or BRCA2 mutation. *New England Journal of Medicine*.

[18] Veronesi A, de Giacomi C, Magri MD (2005). Familial breast cancer: characteristics and outcome of BRCA 1-2 positive and negative cases. *BMC Cancer*.

[19] Kadouri L, Hubert A, Rotenberg Y (2007). Cancer risks in carriers of the BRCA1/2 Ashkenazi founder mutations. *Journal of Medical Genetics*.

[20] Thompson D, Easton DF (2002). Cancer incidence in BRCA1 mutation carriers. *Journal of the National Cancer Institute*.

[21] Cybulski C, Górski B, Gronwald J (2008). BRCA1 mutations and prostate cancer in Poland. *European Journal of Cancer Prevention*.

[22] Suchy J, Cybulski C, Górski B (2010). BRCA1 mutations and colorectal cancer in Poland. *Familial Cancer*.

[23] Easton D (1999). Cancer risks in BRCA2 mutation carriers: the breast cancer linkage consortium. *Journal of the National Cancer Institute*.

[24] Kirchhoff T, Kauff ND, Mitra N (2004). BRCA mutations and risk of prostate cancer in ashkenazi jews. *Clinical Cancer Research*.

[25] Murphy KM, Brune KA, Griffin C (2002). Evaluation of candidate genes MAP2K4, MADH4, ACVR1B, and BRCA2 in familial pancreatic cancer: deleterious BRCA2 mutations in 17%. *Cancer Research*.

[26] Brohet RM, Goldgar DE, Easton DF (2007). Oral contraceptives and breast cancer risk in the international BRCA1/2 carrier cohort study: a report from EMBRACE, GENEPSO, GEO-HEBON, and the IBCCS collaborating group. *Journal of Clinical Oncology*.

[27] Debniak T, Scott RJ, Górski B (2008). Common variants of DNA repair genes and malignant melanoma. *European Journal of Cancer*.

[28] Atchley DP, Albarracin CT, Lopez A (2008). Clinical and pathologic characteristics of patients with BRCA-positive and BRCA-negative breast cancer. *Journal of Clinical Oncology*.

[29] Tung N, Wang Y, Collins LC (2010). Estrogen receptor positive breast cancers in BRCA1 mutation carriers: clinical risk factors and pathologic features. *Breast Cancer Research*.

[30] Loman N, Johannsson O, Bendahl PO, Borg, Fernö M, Olsson H (1998). Steroid receptors in hereditary breast carcinomas associated with BRCA1 or BRCA2 mutations or unknown susceptibility genes. *Cancer*.

[31] Johannsson OT, Idvall I, Anderson C (1997). Tumour biological features of BRCA1-induced breast and ovarian cancer. *European Journal of Cancer A*.

[32] Musolino A, Bella MA, Bortesi B (2007). BRCA mutations, molecular markers, and clinical variables in early-onset breast cancer: a population-based study. *Breast*.

[33] Lee EH, Park SK, Park B (2010). Effect of BRCA1/2 mutation on short-term and long-term breast cancer survival: a systematic review and meta-analysis. *Breast Cancer Research and Treatment*.

[34] Byrski T, Gronwald J, Huzarski T (2010). Pathologic complete response rates in young women with BRCA1-positive breast cancers after neoadjuvant chemotherapy. *Journal of Clinical Oncology*.

[35] Boyd J, Sonoda Y, Federici MG (2000). Clinicopatholic features of BRCA-linked and sporadic ovarian cancer. *Journal of the American Medical Association*.

[36] Piek JMJ, Torrenga B, Hermsen B (2003). Histopathological characteristics of BRCA1- and BRCA2-associated intraperitoneal cancer: a clinic-based study. *Familial Cancer*.

[37] Lacour RA, Westin SN, Meyer LA (2011). Improved survival in non-Ashkenazi Jewish ovarian cancer patients with BRCA1 and BRCA2 gene mutations. *Gynecologic Oncology*.

[38] Chetrit A, Hirsh-Yechezkel G, Ben-David Y, Lubin F, Friedman E, Sadetzki S (2008). Effect of BRCA1/2 mutations on long-term survival of patients with invasive ovarian cancer: the National Israeli Study of Ovarian Cancer. *Journal of Clinical Oncology*.

[39] Hall JM, Lee MK, Newman B (1990). Linkage of early-onset familial breast cancer to chromosome 17q21. *Science*.

[40] Wooster R, Neuhausen SL, Mangion J (1994). Localization of a breast cancer susceptibility gene, BRCA2, to chromosome 13q12-13. *Science*.

[41] Meindl A (2002). Comprehensive analysis of 989 patients with breast or ovarian cancer provides BRCA1 and BRCA2 mutation profiles and frequencies for the German population. *International Journal of Cancer*.

[42] Struewing JP, Hartge P, Wacholder S (1997). The risk of cancer associated with specific mutations of BRCA1 and BRCA2 among Ashkenazi Jews. *New England Journal of Medicine*.

[43] Patel KJ, Yu VPCC, Lee H (1998). Involvement of Brca2 in DNA repair. *Molecular Cell*.

[44] Moynahan ME, Chiu JW, Koller BH, Jasint M (1999). Brca1 controls homology-directed DNA repair. *Molecular Cell*.

[45] Moynahan ME, Pierce AJ, Jasin M (2001). BRCA2 is required for homology-directed repair of chromosomal breaks. *Molecular Cell*.

[46] Scully R, Chen J, Plug A (1997). Association of BRCA1 with Rad51 in mitotic and meiotic cells. *Cell*.

[47] Sharan SK, Morimatsu M, Albrecht U (1997). Embryonic lethality and radiation hypersensitivity mediated by Rad51 in mice lacking Brca2. *Nature*.

[48] Xia F, Taghian DG, DeFrank JS (2001). Deficiency of human BRCA2 leads to impaired homologous recombination but maintains normal nonhomologous end joining. *Proceedings of the National Academy of Sciences of the United States of America*.

[49] Berliner JL, Fay AM (2007). Risk assessment and genetic counseling for hereditary breast and ovarian cancer: recommendations of the National Society of Genetic Counselors. *Journal of Genetic Counseling*.

[50] Evans DGR, Lalloo F, Cramer A (2009). Addition of pathology and biomarker information significantly improves the performance of the Manchester scoring system for BRCA1 and BRCA2 testing. *Journal of Medical Genetics*.

[51] Stopfer JE (2000). Genetic counseling and clinical cancer genetics services. *Seminars in Surgical Oncology*.

[52] Daly MB, Axilbund JE, Buys S (2010). Genetic/familial high-risk assessment: breast and ovarian—clinical practice guidelines in oncology*™*. *Journal of the National Comprehensive Cancer Network*.

[53] U.S. Preventive Services Task Force (2005). Genetic risk assessment and BRCA mutation testing for breast and ovarian cancer susceptibility: recommendation statement. *Annals of Internal Medicine*.

[54] American Congress of Obstetricians and Gynecologists Bulletins (2009). Hereditary breast and ovarian cancer syndrome. *Gynecologic Oncology*.

[55] National Institute for Health and Clinical Excellence Familial breast cancer: the classification and care of women at risk of familial breast cancer in primary, secondary and tertiary care.

[56] Balmaña J, Díez O, Castiglione M (2009). BRCA in breast cancer: ESMO clinical recommendations. *Annals of Oncology*.

[57] Holland ML, Huston A, Noyes K (2009). Cost-effectiveness of testing for breast cancer susceptibility genes. *Value in Health*.

[58] Berg WA, Blume JD, Cormack JB (2008). Combined screening with ultrasound and mammography vs mammography alone in women at elevated risk of breast cancer. *Journal of the American Medical Association*.

[59] Hagen AI, Kvistad KA, Maehle L (2007). Sensitivity of MRI versus conventional screening in the diagnosis of BRCA-associated breast cancer in a national prospective series. *Breast*.

[60] Rijnsburger AJ, Obdeijn I-M, Kaas R (2010). BRCA1-associated breast cancers present differently from BRCA2-associated and familial cases: long-term follow-up of the Dutch MRISC screening study. *Journal of Clinical Oncology*.

[61] Trecate G, Vergnaghi D, Manoukian S (2006). MRI in the early detection of breast cancer in women with high genetic risk. *Tumori*.

[62] Lord SJ, Lei W, Craft P (2007). A systematic review of the effectiveness of magnetic resonance imaging (MRI) as an addition to mammography and ultrasound in screening young women at high risk of breast cancer. *European Journal of Cancer*.

[63] Evans DG, Gaarenstroom KN, Stirling D (2009). Screening for familial ovarian cancer: poor survival of BRCA1/2 related cancers. *Journal of Medical Genetics*.

[64] Vasen HFA, Tesfay E, Boonstra H (2005). Early detection of breast and ovarian cancer in families with BRCA mutations. *European Journal of Cancer*.

[65] Woodward ER, Sleightholme HV, Considine AM, Williamson S, McHugo JM, Cruger DG (2007). Annual surveillance by CA125 and transvaginal ultrasound for ovarian cancer in both high-risk and population risk women is ineffective. *International Journal of Obstetrics and Gynaecology*.

[66] van der Velde NM, Mourits MJE, Arts HJG (2009). Time to stop ovarian cancer screening in BRCA1/2 mutation carriers?. *International Journal of Cancer*.

[67] Goff BA, Mandel LS, Drescher CW (2007). Development of an ovarian cancer symptom index: possibilities for earlier detection. *Cancer*.

[68] Hartmann LC, Sellers TA, Schaid DJ (2001). Efficacy of bilateral prophylactic mastectomy in BRCA1 and BRCA2 gene mutation carriers. *Journal of the National Cancer Institute*.

[69] Rebbeck TR, Friebel T, Lynch HT (2004). Bilateral prophylactic mastectomy reduces breast cancer risk in BRCA1 and BRCA2 mutation carriers: the PROSE study group. *Journal of Clinical Oncology*.

[70] Finch A, Beiner M, Lubinski J (2006). Salpingo-oophorectomy and the risk of ovarian, fallopian tube, and peritoneal cancers in women with a BRCA1 or BRCA2 mutation. *Journal of the American Medical Association*.

[71] Rebbeck TR, Kauff ND, Domchek SM (2009). Meta-analysis of risk reduction estimates associated with risk-reducing salpingo-oophorectomy in BRCA1 or BRCA2 mutation carriers. *Journal of the National Cancer Institute*.

[72] Domchek SM, Friebel TM, Singer CF (2010). Association of risk-reducing surgery in BRCA1 or BRCA2 mutation carriers with cancer risk and mortality. *Journal of the American Medical Association*.

[73] Boughey JC, Hoskin TL, Degnim AC (2010). Contralateral prophylactic mastectomy is associated with a survival advantage in high-risk women with a personal history of breast cancer. *Annals of Surgical Oncology*.

[74] Beattie MS, Crawford B, Lin F, Vittinghoff E, Ziegler J (2009). Uptake, time course, and predictors of risk-reducing surgeries in BRCA carriers. *Genetic Testing and Molecular Biomarkers*.

[75] Friebel TM, Domchek SM, Neuhausen SL (2007). Bilateral prophylactic oophorectomy and bilateral prophylactic mastectomy in a prospective cohort of unaffected BRCA1 and BRCA2 mutation carriers. *Clinical Breast Cancer*.

[76] Kurian AW, Sigal BM, Plevritis SK (2010). Survival analysis of cancer risk reduction strategies for BRCA1/2 mutation carriers. *Journal of Clinical Oncology*.

[77] Kroiss R, Winkler V, Kalteis K (2008). Prevalence of pre-malignant and malignant lesions in prophylactic mastectomy specimens of BRCA1 mutation carriers: comparison with a control group. *Journal of Cancer Research and Clinical Oncology*.

[78] Finch A, Shaw P, Rosen B, Murphy J, Narod SA, Colgan TJ (2006). Clinical and pathologic findings of prophylactic salpingo-oophorectomies in 159 BRCA1 and BRCA2 carriers. *Gynecologic Oncology*.

[79] Hirst JE, Gard GB, Mcillroy K, Nevell D, Field M (2009). High rates of occult fallopian tube cancer diagnosed at prophylactic bilateral salpingo-oophorectomy. *International Journal of Gynecological Cancer*.

[80] Salvador S, Gilks B, Köbel M, Huntsman D, Rosen B, Miller D (2009). The fallopian tube: primary site of most pelvic high-grade serous carcinomas. *International Journal of Gynecological Cancer*.

[81] Greene MH, Mai PL, Schwartz PE (2011). Does bilateral salpingectomy with ovarian retention warrant consideration as a temporary bridge to risk-reducing bilateral oophorectomy in BRCA1/2 mutation carriers?. *American Journal of Obstetrics and Gynecology*.

[82] Fisher B, Costantino JP, Wickerham DL (1998). Tamoxifen for prevention of breast cancer: report of the National Surgical Adjuvant Breast and Bowel Project P-1 study. *Journal of the National Cancer Institute*.

[83] King MC, Wieand S, Hale K (2001). Tamoxifen and breast cancer incidence among women with inherited mutations in brca1 and brca2 national surgical adjuvant breast and bowel project (nsabp-p1) breast cancer prevention trial. *Journal of the American Medical Association*.

[84] Gronwald J, Tung N, Foulkes WD (2006). Tamoxifen and contralateral breast cancer in BRCA1 and BRCA2 carriers: an update. *International Journal of Cancer*.

[85] Duffy SW, Nixon RM (2002). Estimates of the likely prophylactic effect of tamoxifen in women with high risk BRCA1 and BRCA2 mutations. *British Journal of Cancer*.

[86] Metcalfe K, Lynch HT, Ghadirian P (2004). Contralateral breast cancer in BRCA1 and BRCA2 mutation carriers. *Journal of Clinical Oncology*.

[87] Narod SA, Brunet JS, Ghadirian P (2000). Tamoxifen and risk of contralateral breast cancer in BRCA1 and BRCA2 mutation carriers: a case-control study. Hereditary Breast Cancer Clinical Study Group. *The Lancet*.

[88] Metcalfe KA, Birenbaum-Carmeli D, Lubinski J (2008). International variation in rates of uptake of preventive options in BRCA1 and BRCA2 mutation carriers. *International Journal of Cancer*.

[89] Beiner ME, Finch A, Rosen B (2007). The risk of endometrial cancer in women with BRCA1 and BRCA2 mutations. A prospective study. *Gynecologic Oncology*.

[90] Vicus D, Rosen B, Lubinski J (2009). Tamoxifen and the risk of ovarian cancer in BRCA1 mutation carriers. *Gynecologic Oncology*.

[91] Vogel VG, Costantino JP, Wickerham DL (2006). Effects of tamoxifen vs raloxifene on the risk of developing invasive breast cancer and other disease outcomes: the NSABP Study of Tamoxifen and Raloxifene (STAR) P-2 trial. *Journal of the American Medical Association*.

[92] Vogel VG, Costantino JP, Wickerham DL (2007). Erratum: effects of tamoxifen vs raloxifene on the risk of developing invasive breast cancer and other disease outcomes: the NSABP Study of Tamoxifen and Raloxifene (STAR) P-2 trial. *Journal of the American Medical Association*.

[93] Iodice S, Barile M, Rotmensz N (2010). Oral contraceptive use and breast or ovarian cancer risk in BRCA1/2 carriers: a meta-analysis. *European Journal of Cancer*.

[94] Narod SA, Dubé MP, Klijn J (2002). Oral contraceptives and the risk of breast cancer in BRCA1 and BRCA2 mutation carriers. *Journal of the National Cancer Institute*.

[95] Figueiredo JC, Haile RW, Bernstein L (2010). Oral contraceptives and postmenopausal hormones and risk of contralateral breast cancer among BRCA1 and BRCA2 mutation carriers and noncarriers: the WECARE Study. *Breast Cancer Research and Treatment*.

[96] Rebbeck TR, Friebel T, Wagner T (2005). Effect of short-term hormone replacement therapy on breast cancer risk reduction after bilateral prophylactic oophorectomy in BRCA1 and BRCA2 mutation carriers: the PROSE Study Group. *Journal of Clinical Oncology*.

[97] Eisen A, Lubinski J, Gronwald J (2008). Hormone therapy and the risk of breast cancer in BRCA1 mutation carriers. *Journal of the National Cancer Institute*.

[98] Abbas S, Beckmann L, Changclaude J (2010). Polymorphisms in the BRCA1 and ABCB1 genes modulate menopausal hormone therapy associated breast cancer risk in postmenopausal women. *Breast Cancer Research and Treatment*.

[99] Turner N, Tutt A, Ashworth A (2004). Hallmarks of ‘BRCAness’ in sporadic cancers. *Nature Reviews Cancer*.

[100] Bozzetti C, Bortesi B, Merisio C (2004). Loss of heterozygosity (LOH) in ovarian cancer. *International Journal of Gynecology and Obstetrics*.

[101] Birgisdottir V, Stefansson OA, Bodvarsdottir SK, Hilmarsdottir H, Jonasson JG, Eyfjord JE (2006). Epigenetic silencing and deletion of the BRCA1 gene in sporadic breast cancer. *Breast Cancer Research*.

[102] Weberpals JI, Clark-Knowles KV, Vanderhyden BC (2008). Sporadic epithelial ovarian cancer: clinical relevance of brca1 inhibition in the DNA damage and repair pathway. *Journal of Clinical Oncology*.

[103] Thrall M, Gallion HH, Kryscio R, Kapali M, Armstrong DK, Deloia JA (2006). BRCA1 expression in a large series of sporadic ovarian carcinomas: a Gynecologic Oncology Group study. *International Journal of Gynecological Cancer*.

[104] Weberpals J, Garbuio K, O'Brien A (2009). The DNA repair proteins BRCA1 and ERCC1 as predictive markers in sporadic ovarian cancer. *International Journal of Cancer*.

[105] Lambie H, Miremadi A, Pinder SE (2003). Prognostic significance of BRCA1 expression in sporadic breast carcinomas. *Journal of Pathology*.

[106] Yang Q, Sakurai T, Mori I (2001). Prognostic significance of BRCA1 expression in Japanese sporadic breast carcinomas. *Cancer*.

[107] Taron M, Rosell R, Felip E (2004). BRCA1 mRNA expression levels as an indicator of chemoresistance in lung cancer. *Human Molecular Genetics*.

[108] Margeli M, Cirauqui B, Castella E (2010). The prognostic value of BRCA1 mRNA expression levels following neoadjuvant chemotherapy in breast cancer. *PLoS ONE*.

[109] Quinn JE, James CR, Stewart GE (2007). BRCA1 mRNA expression levels predict for overall survival in ovarian cancer after chemotherapy. *Clinical Cancer Research*.

[110] Weberpals JI, Tu D, Squire JA Breast cancer 1 (BRCA1) protein expression as a prognostic marker in sporadic epithelial ovarian carcinoma: an NCIC CTG OV.16 correlative study 5.

[111] Helleday T, Petermann E, Lundin C, Hodgson B, Sharma RA (2008). DNA repair pathways as targets for cancer therapy. *Nature Reviews Cancer*.

[112] Fong PC, Boss DS, Yap TA (2009). Inhibition of poly(ADP-ribose) polymerase in tumors from BRCA mutation carriers. *New England Journal of Medicine*.

[113] Tutt A, Robson M, Garber JE (2010). Oral poly(ADP-ribose) polymerase inhibitor olaparib in patients with BRCA1 or BRCA2 mutations and advanced breast cancer: a proof-of-concept trial. *The Lancet*.

